# An Atypical Presentation of Emphysematous Pyelonephritis Complicated by Methicillin-Resistant Staphylococcus aureus Bacteremia

**DOI:** 10.7759/cureus.50721

**Published:** 2023-12-18

**Authors:** Matthew Carvey, Matthew Roehrs

**Affiliations:** 1 Emergency Medicine, Cleveland Clinic, Cleveland, USA; 2 Emergency Medicine, MetroHealth Medical Center, Cleveland, USA

**Keywords:** urinary tract obstruction, diabetes mellitus, sepsis, mrsa bacteremia, emphysematous pyelonephritis

## Abstract

Emphysematous pyelonephritis is an acute necrotizing infection of the renal parenchyma, collecting system, and surrounding perinephric tissue, characterized by the presence of gas within these locations on imaging. It is associated with high mortality rates and is often found in diabetic patients. We present the case of a 60-year-old female, with a past history of Von Willebrand disease and hypertension, who presented to our emergency department complaining of acute-on-chronic right knee, left hip, and paraspinal lumbar back pain with an increased frequency of falling for approximately one week. She was found to have pursed-lip breathing on physical exam. Due to her vague clinical presentation, this condition was incidentally discovered on initial workup for a pulmonary embolus, found to have extensive air in the left renal collecting system on CT of the chest. Broad-spectrum antibiotics and monitoring of hemodynamics were part of the initial resuscitation with eventual percutaneous nephrostomy tube placement by interventional radiology. Post-operatively, she developed acute respiratory distress syndrome further complicated by methicillin-resistant *Staphylococcus aureus* bacteremia. It is important to keep emphysematous pyelonephritis in the differential even in the absence of risk factors and signs of urosepsis when a patient presents with vague signs and symptoms of this disease.

## Introduction

Emphysematous pyelonephritis (EP) is a rare condition most commonly associated with poor glycemic control in patients with diabetes mellitus who develop an acute renal infection. Urinary tract obstructions have also been reported as a common cause of EP. Emphysematous pyelitis, on the contrary, is a more benign form of the condition involving only the collecting system, and often secondary to iatrogenic interventions or urinary tract obstructions. Patients who present with EP generally complain of flank pain, renal angle tenderness, and a fever. Here we present an atypical case of emphysematous pyelonephritis complicated by Methicillin-resistant *Staphylococcus aureus* (MRSA) bacteremia and a staghorn calculus in a 60-year-old female without a prior diagnosis of diabetes mellitus or urinary tract obstructions.

## Case presentation

A 60-year-old non-diabetic woman living in the United States presented to the emergency department with a past history of Von Willebrand's disease and hypertension. On presentation, she expressed the motivation behind the visit was to investigate her acute-on-chronic right knee, left hip, and paraspinal lumbar back pain, which had progressively worsened over the last week leading to a fall. Her initial vital signs were stable with a blood pressure of 131/75, heart rate of 96, respiratory rate of 20, oxygen saturation of 95% on room air, and temperature of 98.2°F.

The routine examination was unremarkable other than pursed-lip breathing and palpable tenderness to the right knee and left hip, without the patient feeling short of breath or dyspneic on exertion. She denied a prior history of diabetes mellitus with a recent hemoglobin A1C of 5.1%, recent urinary tract infections, chronic Foley catheter requirements, associated rashes or skin findings, intravenous drug or alcohol use, cigarette smoking, recent sexual intercourse, international travel, or kidney stones. Family history was remarkable only for Von Willebrand's disease. 

Although the patient presented with a vague history and physical exam, further investigation into the cause of her pursed-lip breathing was necessary. Initial laboratory results are presented in Table 1.

**Table 1:** Laboratory results on initial presentation.

Laboratory value	Result (SI)	Reference range (SI)
White blood cell	35.1 x 10^9^/liter	4.5-11 x 10^9^/liter
Hemoglobin	8.75 mmol/L	7.4-9.9 mmol/L
Hematocrit	0.434 fraction of RBC’s	0.36-0.46 fraction of RBC’s
Platelets	137 x 10^9^/L	130-400 x 10^9^/L
Sodium	140 mEq/L	135-145 mEq/L
Potassium	3.2 mEq/L	3.4-5.0 mEq/L
Chloride	103 mEq/L	95-108 mEq/L
Carbon dioxide	20 mmol/L	20-32 mmol/L
Blood urine nitrogen	17.1 mmol/liter	2.9-8.9 mmol/liter
Creatinine	123.76 umol/L	44-97 umol/L
Blood glucose	5.16 mmol/L	3.9-6.1 mmol/L

**Figure 1:**
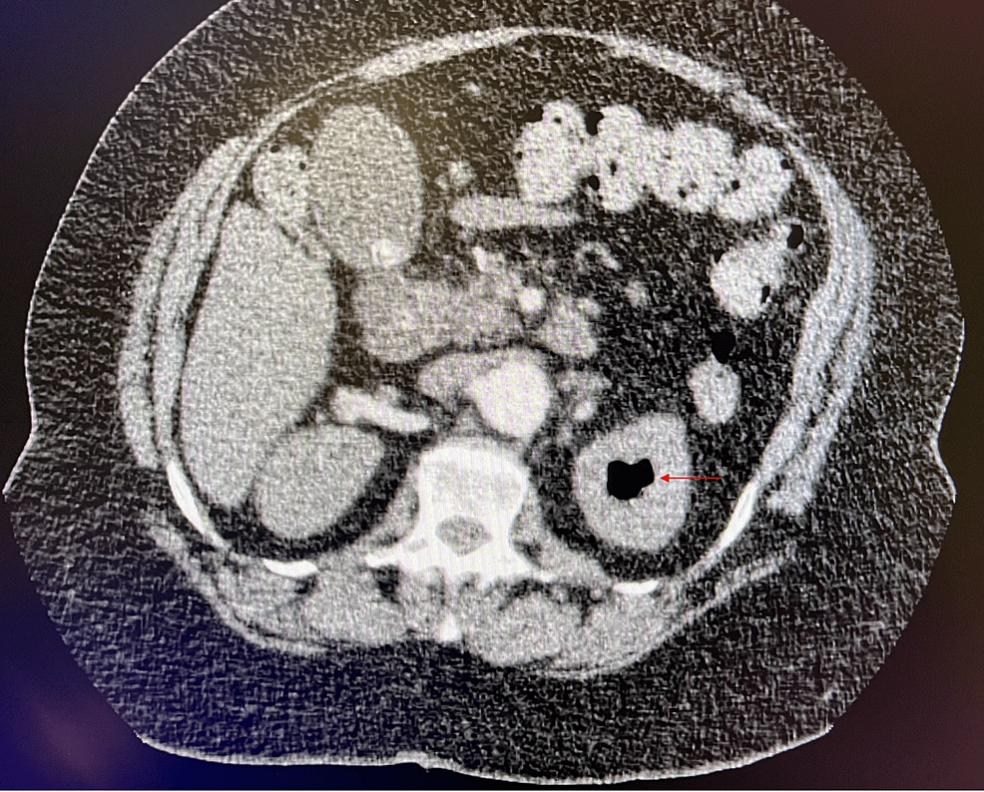
Computer tomography of the chest showing extensive air in the left renal collecting system.

A chest X-ray was also obtained. Attempts to collect a urinalysis were unsuccessful in the emergency department due to urinary retention. In the presence of an elevated d-dimer at 4,036ug/L DDU (D-dimer units), mildly elevated troponin, and pursed-lip breathing, a CT of the chest with contrast was ordered to rule out an acute pulmonary embolus. CT scanning of the chest was unremarkable for a pulmonary embolus; however, there was concern for extensive air in the left renal collecting system and bilateral staghorn calculi (Figure 1).

**Figure 2:**
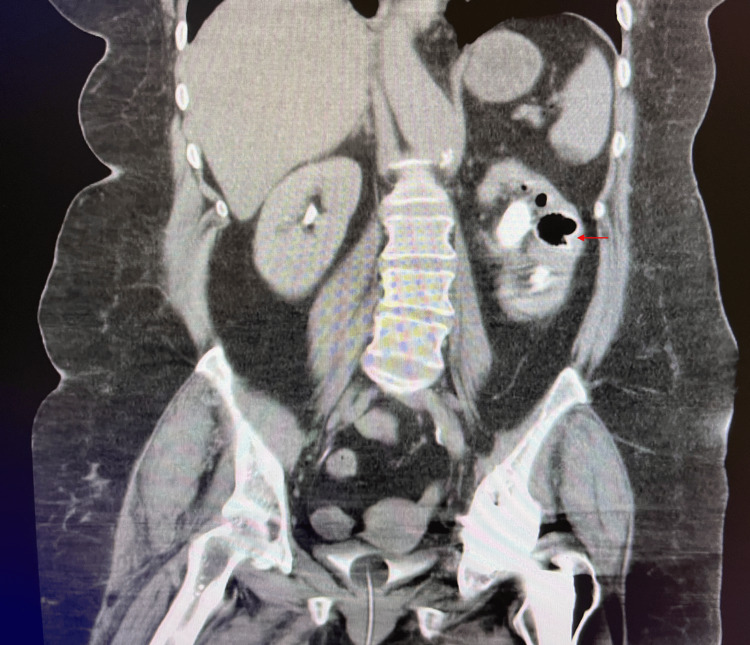
Computer tomography of the abdomen and pelvis showing bilateral renal staghorn calculi, with the left renal staghorn calculus displaying suspicious components of possible intraparenchymal gas locules.

This prompted further radiographic imaging with a CT abdomen and pelvis with contrast to rule out the suspicion of emphysematous pyelitis by radiology. CT scanning of the abdomen and pelvis exposed a prominent left renal staghorn calculus with suspicious components of possible intraparenchymal gas locules, revealing a likely diagnosis of emphysematous pyelonephritis (Figure 2). The broad-spectrum antibiotics vancomycin and Zosyn were initiated and the patient was taken to the operating room emergently by urology and interventional radiology after becoming tachycardic and febrile, displaying early signs of shock.

Intra-operatively, the patient underwent percutaneous nephrostomy tube placement. Post-operatively, a urinalysis and urine culture were collected on the inpatient unit once the patient was able to produce urine, which revealed a large amount of blood, positive leukocyte esterase, and an *Escherichia coli* urinary tract infection. While in the intensive care unit, the patient developed acute respiratory distress syndrome (ARDS) requiring prolonged intubation. Her condition was further complicated by MRSA bacteremia found on blood culture analysis, presumed to be secondary to the staghorn calculi, necessitating further escalation in the antibiotic regimen to ertapenem. An echocardiogram was completed on findings of MRSA bacteremia, returning negative for valvular vegetations. She was eventually extubated and discharged after an extended ICU course with outpatient monitoring of her staghorn calculi, continued management of her percutaneous nephrostomy, and tentative plans for surgical stone removal in three months. She recovered well post-discharge and has not required readmission.

## Discussion

EP is a rare condition, seen only one to two times per year in a typical busy urological department in the United States [1]. Given its rare presentation, the diagnosis can be challenging, leading to its potentially high mortality rate if there are delays in early intervention.

EP has been defined as a necrotizing infection of the renal parenchyma, collecting system, or perinephric tissue [2-8]. To date, diabetes mellitus is the most common comorbidity associated with EP, affecting 70% to 90% of patients [9]. The risk of developing EP secondary to a urinary tract obstruction is about 25-40%, with *Escherichia coli* being the most common pathogen isolated from the urine or pus culture [10]. EP also disproportionately affects females, with a female-to-male ratio of 3:1 [11].

The true pathophysiology behind EP is still unclear; however, the primary hypothesis is that intra-renal fermentation of glucose in the presence of anaerobic Gram-negative bacteria creates a suitable environment to produce gas, causing decreased tissue perfusion and eventual necrotizing tissue infections [12]. A second hypothesis suggests obstructive uropathies form a nidus for infection, causing stagnation or reflux of urine with a lack of laminar flow in the ureteral system and a resultant ascending infection [13]. Therefore, prompt recognition of EP is necessary to prevent the rapid progression of this disease. 

The diverse, and in this case atypical, clinical presentation of EP can often lead to delayed management. Common differential diagnoses include focal renal abscess, accidental or iatrogenic trauma to the kidney, or enterorenal fistula [14]. In a meta-analysis by Ngo et al., mortality among patients with EP was found to be 13%, which is a significant improvement from prior years largely due to minimally invasive interventions and a decreased emphasis on emergency nephrectomies [15]. However, concomitant sepsis, acute renal failure, altered mental status or shock have a negative impact on the survivability of EP. For comparison, there are few cases of EP complicated by a separate MRSA bacteremia, with those cited in the literature stating the patient had a prior history of diabetes mellitus. Thus, special consideration into the specific antibiotic regimen should be measured to adequately treat the underlying infection, with the utmost importance being on early recognition of EP to ensure timely surgical involvement.

The most common signs and symptoms of EP are fever and pyuria, followed by flank pain, tachycardia, and renal angle tenderness [16]. However, a critically ill patient without these symptoms with a past medical history of diabetes mellitus or previously diagnosed with a urinary tract obstruction should still prompt further workup for EP. A kidney, ureter, and bladder (KUB) X-ray and ultrasound are initial diagnostic modalities to consider, but an abdominal CT is the most sensitive imaging study for diagnosing EP [17,18].

Early management of EP includes hemodynamic resuscitation, broad-spectrum antibiotics, glycemic control, and surgical intervention [19]. Third-generation cephalosporins, aminoglycosides, and fluoroquinolones are first-line antimicrobials for conservative management of EP. In cases of EP complicated by MRSA bacteremia and septic shock, conservative antibiosis strategies should include vancomycin to cover this species [20]. Urgent urology consultation and percutaneous drainage, followed by delayed nephrectomy in the case of a nonfunctioning kidney, is presently the gold standard for the management of EP in conjunction with conservative management [17].

In our case presented above, this 60-year-old female presented with pursed-lip breathing and vague complaints of acute-on-chronic knee, back, and hip pain with an increased frequency of falling. She was initially worked up with a CT scan of the chest to rule out an acute pulmonary embolus, where emphysematous pyelitis and eventual emphysematous pyelonephritis were diagnosed on a CT scan of the abdomen and pelvis. She was taken emergently to the operative room for percutaneous nephrostomy tube placement. The patient denied any history of diabetes mellitus or urinary tract obstructions.

## Conclusions

We present a rare case of emphysematous pyelonephritis complicated by methicillin-resistant *Staphylococcus aureus* (MRSA) bacteremia. The historical absence of diabetes mellitus or urinary tract obstructions and vague chronic complaints can lead to a delayed or missed diagnosis. The case we present above is that of a 60-year-old female who presented to the emergency department with pursed-lip breathing and acute-on-chronic back pain, later found to be caused by emphysematous pyelonephritis. She was treated with broad spectrum antibiotics and percutaneous nephrostomy tube placement, eventually complicated by MRSA bacteremia and acute respiratory distress syndrome. Therefore, it is important to broaden the differential to include abdominal and urological pathologies in ill-appearing patient’s with vague symptoms, with early diagnosis and treatment being needed to prevent progression of emphysematous pyelonephritis.

## References

[1] Jakle H, Winter A, Pena N (2017). Massive emphysematous pyelonephritis. Clin Pract Cases Emerg Med.

[2] Michaeli J, Mogle P, Perlberg S, Hemiman S, Caine M (1984). Emphysematous pyelonephritis. J Urol.

[3] Wan YL, Lee TY, Bullard MJ, Tsai CC (1996). Acute gas-producing bacterial renal infection: correlation between imaging findings and clinical outcome. Radiology.

[4] Godec CJ, Cass AS, Berkseth R (1980). Emphysematous pyelonephritis in a solitary kidney. J Urol.

[5] DePauw AP, Ross G (1981). Emphysematous pyelonephritis in a solitary kidney. J Urol.

[6] Hudson MA, Weyman PJ, van der Vliet AH, Catalona WJ (1986). Emphysematous pyelonephritis: successful management by percutaneous drainage. J Urol.

[7] Paivansalo M, Hellstrom P, Siniluoto T, Leinonen A (1989). Emphysematous pyelonephritis: radiologic and clinical findings in six cases. Acta Radiol.

[8] Gold RP, McClennan BL, Kenney PJ (1990). Acute infections of the renal parenchyma. Clinical Urography.

[9] Arrambide-Herrera JG, Robles-Torres JI, Ocaña-Munguía MA, Romero-Mata R, Gutiérrez-González A, Gómez-Guerra LS (2022). Predictive factors for mortality and intensive care unit admission in patients with emphysematous pyelonephritis: 5-year experience in a tertiary care hospital. Actas Urol Esp (Engl Ed).

[10] Ubee SS, McGlynn L, Fordham M (2011). Emphysematous pyelonephritis. BJU Int.

[11] Wan YL, Lo SK, Bullard MJ, Chang PL, Lee TY (1998). Predictors of outcome in emphysematous pyelonephritis. J Urol.

[12] Dutta P, Bhansali A, Singh SK, Gupta KL, Bhat MH, Masoodi SR, Kumar Y (2007). Presentation and outcome of emphysematous renal tract disease in patients with diabetes mellitus. Urol Int.

[13] Karthikeyan VS, Manohar CM, Mallya A, Keshavamurthy R, Kamath AJ (2018). Clinical profile and successful outcomes of conservative and minimally invasive treatment of emphysematous pyelonephritis. Cent European J Urol.

[14] Sureka B, Thukral BB (2012). Emphysematous infections of the urinary tract: a radiological perspective. Indian J Nephrol.

[15] Ngo XT, Nguyen TT, Dobbs RW (2022). Prevalence and risk factors of mortality in emphysematous pyelonephritis patients: a meta-analysis. World J Surg.

[16] Desai R, Batura D (2022). A systematic review and meta-analysis of risk factors and treatment choices in emphysematous pyelonephritis. Int Urol Nephrol.

[17] Somani BK, Nabi G, Thorpe P, Hussey J, Cook J, N'Dow J (2008). Is percutaneous drainage the new gold standard in the management of emphysematous pyelonephritis? Evidence from a systematic review. J Urol.

[18] Huang JJ, Tseng CC (2000). Emphysematous pyelonephritis: clinicoradiological classification, management, prognosis, and pathogenesis. Arch Intern Med.

[19] Kapoor R, Muruganandham K, Gulia AK (2010). Predictive factors for mortality and need for nephrectomy in patients with emphysematous pyelonephritis. BJU Int.

[20] Rafiq N, Nabi T, Rasool S, Sheikh RY (2021). A prospective study of emphysematous pyelonephritis in patients with type 2 diabetes. Indian J Nephrol.

